# Effect of Diets Differing in Glycemic Index and Glycemic Load on Cardiovascular Risk Factors: Review of Randomized Controlled-Feeding Trials

**DOI:** 10.3390/nu5041071

**Published:** 2013-03-28

**Authors:** Aleksandra S. Kristo, Nirupa R. Matthan, Alice H. Lichtenstein

**Affiliations:** Cardiovascular Nutrition Laboratory, Jean Mayer USDA Human Nutrition Research Center on Aging, Tufts University, Boston, MA 02111, USA; E-Mails: aleksandra.kristo@tufts.edu (A.S.K.); nirupa.matthan@tufts.edu (N.R.M.)

**Keywords:** glycemic index, GI, glycemic load, GL, controlled-feeding trial, cardiovascular disease, CVD, glucose, lipids, inflammation

## Abstract

Despite a considerable amount of data available on the relationship between dietary glycemic index (GI) or load (GL) and cardiovascular disease (CVD) risk factors, in aggregate, the area remains unsettled. The aim of the present review was to summarize the effect of diets differing in GI/GL on CVD risk factors, by examining randomized controlled-feeding trials that provided all food and beverages to adult participants. The studies included a low and high GI/GL diet phase for a minimum of four weeks duration, and reported at least one outcome related to CVD risk; glucose homeostasis, lipid profile or inflammatory status. Ten publications representing five trials were identified. The low GI/GL compared to the high GI/GL diet unexpectedly resulted in significantly higher fasting glucose concentrations in two of the trials, and a lower area under the curve for glucose and insulin in one of the two studies during an oral glucose tolerance test. Response of plasma total, low density lipoprotein and high density lipoprotein cholesterol concentrations was conflicting in two of the studies for which data were available. There was either weak or no effect on inflammatory markers. The results of the five randomized controlled trials satisfying the inclusion criteria suggest inconsistent effects of the GI/GL value of the diet on CVD risk factors.

## 1. Introduction

Although it is well recognized that carbohydrate-containing foods elicit different postprandial glucose responses, the factors that influence this variation have yet to be fully elucidated [1,2]. In an attempt to describe the post ingestion effect of different carbohydrate containing foods on blood glucose concentrations, Jenkins *et al*. [3], coined the term “glycemic index” (GI), defined as the relative area under the curve for blood glucose concentrations during a two-hour period after consuming a test food compared to a standard food (glucose or white bread) containing the same amount of digestible carbohydrate (50 g) [3]. A companion term, glycemic load (GL), is calculated by adjusting the GI value of the food to the serving size as quantified by carbohydrate content [4]. This classification system has been promulgated for use as a tool to guide food choices to reduce chronic disease risk. However, considerable controversy exists about the utility of supplementing current population-based dietary recommendations with specific guidance for the GI or GL value of foods, particularly with respect to improving cardiovascular disease (CVD) risk factors [5,6,7,8].

In an attempt to address this issue, Opperman *et al.* [9], performed a meta-analysis summarizing randomized controlled trials (RCT’s) published between 1981 and 2003 that compared low to high GI diets [9]. For inclusion into the meta-analysis the studies had to either provide key or all foods, or instruct the subjects on how to choose food on the basis of GI values. They concluded that low GI compared to high GI diets significantly improved markers of glycemic control (fructosamine; glycosylated hemoglobin, HbA1c) and total cholesterol concentration. However, the GI value of the diets had no significant effect on high density lipoprotein (HDL) cholesterol, low density lipoprotein (LDL) cholesterol, or triglyceride (TG) concentrations. In some participant subgroups, for example, individuals with diabetes, a non significant trend towards lower LDL cholesterol concentrations was noted.

A Cochrane meta-analysis, first published in 2004 and updated in 2006, identified 21 RCT’s that included data for the effects of GI interventions on coronary heart disease (CHD) risk factors [10]. Inclusion criteria were adult participants with at least one major CHD risk factor or a CHD diagnosis, who were provided with either dietary instruction or food for a minimum of a four week intervention period. On the basis of sensitivity analysis, conducted when included studies contributed a significant amount to the pooled results, the authors concluded that there was no significant effect of the GI value of the diet on fasting glucose, insulin, HbA1c, HDL cholesterol and TG concentrations. They further concluded that the low GI diets compared to the high GI diets resulted in a modest lowering of LDL cholesterol concentration (−0.16 mmol/L, 95% CI −0.32 to 0.00, *p* = 0.05). The author’s final assessment was that “any beneficial effect of low GI diets on CHD and its risk factors is small”. A subsequent review with similar inclusion criteria came to same conclusions [6]. 

In contrast, a more recent meta-analysis summarizing RCT’s published through March 2012 comparing studies that provided at least one meal per day with a low and high GI value reported significantly lower total and LDL cholesterol concentrations for the low GI relative to the high GI diet phase, but no significant effect of dietary GI on HDL cholesterol or TG concentrations [11].

Of note, most of the reviews on the topic available thus far did not differentiate between RCT’s for which participants were provided with instruction to modify the GI value of their diet or only provided with a limited number of foods compared to RCT’s that used a feeding protocol in which all food and beverage were provided. The latter experimental design minimizes potential differences among study participants with regard to adherence to the dietary protocol. This brief review is focused on RCT’s that stated use of a controlled-feeding protocol in which all food and beverage were provided and compared the effect of low and high GI/GL diets with comparable macronutrient distributions on CVD risk factors. 

## 2. Methods

### 2.1. Search Strategy

**Figure 1:**
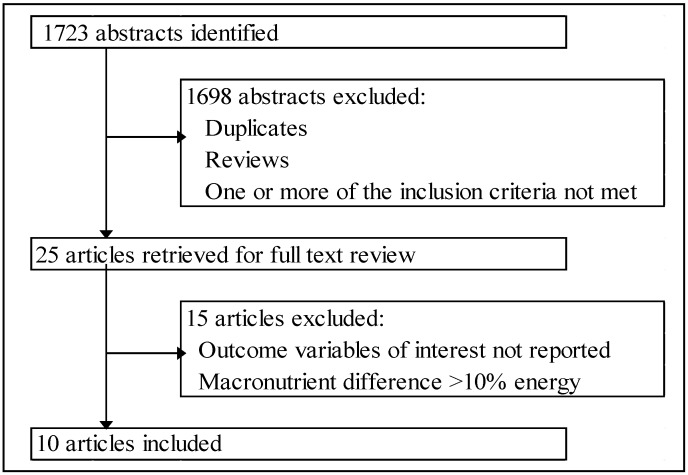
Flow chart of study selection.

A Pubmed advanced search was performed through 15 December 2012 using the words “glycemic” (medical subject heading, MeSH terms or title/abstract) or “glycaemic” (title/abstract) in the following combinations: glycemic (or glycaemic), glycemic (or glycaemic) index, low-glycemic (or glycaemic), high-glycemic (or glycaemic) and diet (MeSH terms or title/abstract) applying limitations (filters) for article types (clinical trials or randomized controlled trials), date of publication (from 1 January 2002 to 30 December 2012), species (humans, MeSH terms), ages (adult +19, MeSH terms), and language (English). Abstracts were screened and for those that appeared to meet the inclusion criteria full texts of the articles were retrieved and further screened. Figure 1 summarizes the search results. Reference lists of recent review articles were also screened for additional published work. 

### 2.2. Inclusion Criteria

Inclusion criteria were: (1) controlled human trials with all foods and beverages provided throughout the study period; (2) a low and high GI or GL diet phase with comparable macronutrient compositions (≤10 percent energy difference); (3) minimum four week intervention per diet phase; (4) adult participants (age ≥19 years); (5) data for at least one variable related to glucose homeostasis, blood lipids or inflammation; and (6) full text available in English. 

## 3. Results

### 3.1. Included Studies

Five individual trials generating data presented in ten published manuscripts met the inclusion criteria. These included one cross-over trial focusing on GI and GL [12], one cross-over trial with two publications [13,14] focusing on GI, one cross-over trial with three publications focusing on GL [15,16,17], one parallel trial with a single publication [18] and one parallel trial with three publications [19,20,21], both parallel trials focusing on GI. The cross-over trials were designed to maintain constant body weight, while the parallel trials were intended to induce weight loss by reducing energy intake [18], or increasing energy expenditure while maintaining constant energy intake [19,20,21]. In two publications the study population included a subset of the parent trial [16,21]. The study designs and diet compositions are summarized in Table 1, Table 2, respectively. 

**Table 1:** Summary of study design and participant characteristics.

Trial	Reference	Design	Intervention	Duration, Weeks (Washout)	Sex ( *n*)	Participants	Age, Years, Mean (SD) or Range	BMI (SD)
**1**	[12]	R-X	LGI/GL-HGI/GL	4 (4)	M (24)	Ob	34.5 (8.1)	29.5 (4.3)
**2**	[13]	R-X	LGI-HGI	4 (~2)	M (64)	IS + IR	54.5 (7.8)	28.7 (3.5)
**2**	[14]	R-X	LGI-HGI	4 (~2)	M (64)	IS + IR	53.5 (7.6), IS	27.4 (3.2), IS
							55.5 (8.0), IR	30.3 (3.2), IR
**3**	[15]	R-X	LGL-HGL	4 (4)	M (40), F (40)	NW + OW/Ob	29.6 (8.2)	27.4 (5.9)
**3**	[16]	R-X	LGL-HGL	4 (4)	M (9), F (7)	NW + OW/Ob	19–44	18.5–25, NW
								28–40, OW/Ob
**3**	[17]	R-X	LGL-HGL	4 (4)	M (40), F (40)	NW + OW/Ob	18–45	27.5 (5.9)
**4**	[18]	R-P	LGI-HGI	12	M (5), F (14)	Ob	18–70	30–40
**5**	[19]	R-P	(LGI + E)-(HGI + E)	12	M (8), F (14)	Ob + prediabetic	66 (1)	34.4 (2.8)
**5**	[20]	R-P	(LGI + E)-(HGI + E)	12	M (13), F (15)	Ob + IR	66 (1)	34.2 (0.7)
**5**	[21]	R-P	(LGI + E)-(HGI + E)	12	M (11), F (10)	MetS	66.2 (1.1)	35.3 (0.9)

Numbers in the first column indicate unique trials; publications with the same number indicate same trial; SD, standard deviation; BMI, body mass index; R-X, randomized crossover; R-P, randomized parallel; LGI/GL or HGI/GL, low or high glycemic index/glycemic load diet; E, exercise; M, male; F, female; Ob, obese; IS, insulin sensitive; IR, insulin resistant; NW, normal weight; OW, overweight; MetS, metabolic syndrome.

**Table 2:** Composition of low and high GI/GL diets by study.

Trial	Reference	GI		GL		Carbohydrate		Fat		Protein		Fiber	
								%Energy					
		low	high	low	high	low	high	low	high	low	high	low	high
**1**	[12]	50	75	158	246	55	56	29	30	18	16	23 ^a^	21 ^a^
**2**	[13,14]	38	69	84	152	50	50	34	34	18	18	21 ^b^	9 ^b^
**3**	[15,16,17]	34	78	≤125	≥250	55	55	30	30	15	15	49 ^a^	24 ^a^
**4**	[18]	33	63	178	272	60	60	25	25	15	15	17 ^b^	9 ^b^
**5**	[19,20,21]	40	80	102	218	56	58	32	32	17	17	28 ^a^	28 ^a^

Numbers in the first column indicate unique trials; GI, glycemic index; GL, glycemic load; ^a^ g/day; ^b^ g/1000 kcal or g/4184 kJ.

### 3.2. Outcome Variables

#### 3.2.1. Glucose Homeostasis

*Cross-Over Studies*—Inconsistent data were reported for the effect of dietary GI values on fasting glucose concentrations. Of the four studies identified [12,13,15,16], two reported no significant effect [12,16] and two reported significantly higher fasting glucose concentrations after subjects consumed the low GI compared to high GI diet [13,15] (Table 3). 

**Table 3:** Effect of low *versus* high GI/GL diets on selected glucose homeostasis markers, lipid profile and inflammatory markers.

Trial	Reference	Participants	Glucose Homeostasis Markers					Lipid Profile				Inflammatory Markers		
			Glc	Ins	%HbA1c	Glc OGTT	Ins OGTT	TC	LDL	HDL	TG	CRP	IL-6	TNFα
*Cross-Over Studies*														
**1**	[12]	Ob	↔	↔	–	–	–	↑	↑	↑	↔	↔	↔	↔
**2**	[13]	IS/IR	↑	↔	–	–	–	–	–	–	–	↔	–	–
		IS	↔	↔								↔		
		IR	↑	↔								↔		
**2**	[14]	IS/IR	–	–	–	–	–	↓	↓	↓	↔	–	–	–
		IS						↓	↓	↔	↔			
		IR						↔	↓	↓	↔			
**3**	[15]	LBF/HBF	↑	↔	–	–	–	–	–	–	–	–	–	–
		LBF	↔	↔										
		HBF	↑	↔										
**3**	[16]	LBF/HBF	↔	↔	–	–	–	–	–	–	–	–	–	–
		LBF	↔	↔										
		HBF	↔	↔										
**3**	[17]	LBF/HBF	–	–	–	–	–	–	–	–	–	↔	↔	–
		LBF										↔	↑	
		HBF										↓	↔	
*Parallel Studies*														
**4**	[18]	Ob	↔	↔	–	–	–	–	–	–	↔	–	–	–
**5**	[19]	Ob	↔	↔	↔	↓	↓	↔	↔	↔	↔	–	–	–
**5**	[20]	Ob	↔	↔	↔	↔	↔	–	–	–	–	–	↓	↓
**5**	[21]	Ob	↔	↔	↔	–	–	↔	↔	↔	↔	–	–	–

Numbers in the first column indicate unique trials; publications with the same number indicate same trial; Glc, glucose; Ins, insulin; HbA1c, glycosylated hemoglobin; OGTT, oral glucose tolerance test; TC, total cholesterol; LDL, low density lipoprotein cholesterol; HDL, high density lipoprotein cholesterol; TG, triglycerides; CRP, C-reactive protein; IL-6, interleukin-6; TNFα, tumor necrosis factor alpha; ↑ or ↓, significantly increased or decreased compared to high GI/GL diet; ↔, non significant effect of low compared to high GI/GL diet; –, not reported; Ob, obese; IS, insulin sensitive; IR, insulin resistant; LBF, low body fat; HBF, high body fat.

None of the four studies that assessed fasting insulin concentrations reported a significant effect of the dietary GI or GL value [12,13,15,16] (Table 3). None of the cross-over studies measured HbA1c, or performed OGTT. With regard to additional glucose homeostasis variables (Table S1), the concentrations of fasting C-peptide, and the incretins glucagon-like peptide (GLP-1) and glucose-dependent insulinotropic polypeptide (GIP) were unaffected by the GI values of the diets [13,16]. In a single study for which data are available, the low GL diets compared to the high GL diet resulted in lower insulin-like growth factor-1 (IGF-1) concentrations [15] (Table S1). Of note, in the postprandial state, the lower GL compared to the higher GL diet resulted in a higher incremental area under the curve (iAUC) for GLP-1, but lower iAUC for GIP (Table S3) [16].

*Parallel Studies*—None of the four studies using a parallel design reported a significant difference in fasting plasma glucose or insulin concentrations between the groups consuming diets with two different GI values [18,19,20,21]. Similar findings were reported for the three studies that also assessed HbA1c concentrations [19,20,21]. Of the two studies that reported data for glucose and insulin AUC after oral glucose tolerance test (OGTT), one study documented a lower AUC for both variables in the group provided with the low GI diet than the high GI diet [19], while the other found no significant effect of dietary GI [20] (Table 3). Fasting C-peptide concentrations was similar between groups, whereas C-peptide and GIP concentrations measured during the OGTT were lower in the participants provided with the low compared to high GI diets [19] (Table S1). In a single study that conducted a euglycemic/hyperinsulinemic clamp procedure a greater reduction in insulin secretion rate (ISR) was reported for the group provided with the low GI diet compared to high GI diet [19] (Table S1). Additional variables measured during OGTT and euglycemic clamp, such as glucose and insulin concentrations at two hours post glucose administration, plasma non-esterified fatty acids (NEFA) and substrate utilization, were unaffected by the GI value of the diet [19,20,21] (Table S1). 

#### 3.2.2. Lipid Profile

*Cross-Over Studies*—Of the two studies that reported total, LDL and HDL cholesterol concentrations contradictory findings were reported, one found a significant increase [12] and the other a significant decrease with the low GI compared to high GI diet for all three variables [14] (Table 3). The type of diet had no significant effect on the TG concentrations in the two studies reporting these data [12,14] (Table 3). 

*Parallel Studies—*When data were available total, LDL, HDL and very low density lipoprotein (VLDL) cholesterol, and TG and concentrations were unaffected by the GI value of the diets [18,19,21] (Table 3, Table S2). 

#### 3.2.3. Inflammatory Markers

*Cross-Over Studies*—C-reactive protein (CRP), interleukin-6 (IL-6) and tumor necrosis alpha (TNFα) concentrations were not significantly affected by the GI value of the diets in all studies for which data were available [12,13,17] (Table 3). Similarly, TNFα receptors (I and II) and serum amyloid A (SAA) were not significantly affected by the GI value of the diet (Table S2). 

*Parallel Studies*—In the single study for which inflammatory data are available lower IL-6 and TNFα concentrations were reported for the group receiving the low GI compared to the high GI diet [20] (Table 3). Additionally, the low GI diet resulted in a lower TNFα *ex vivo* secretion from mononuclear cells (MNC) (Table S2). MNC secretion of IL-6 from or monocyte chemoattractant protein 1 (MCP-1) concentrations were not significantly affected by the dietary GI value (Table S2).

#### 3.2.4. Additional Variables

No significant effects of the GI/GL value of the diet were reported for plasminogen activator inhibitor 1 (PAI-1), fibrinogen, leptin or adiponectin (Table S2).

## 4. Discussion

The current review summarizes the five available controlled-feeding trials comparing the effect of low and high GI/GL diets on selected indicators of CVD risk related to glucose homeostasis, blood lipids and inflammatory status. Three of these trials were cross-over and designed for maintaining weight and two were parallel trials designed for weight loss by reducing energy intake [18], or maintaining constant energy intake while increasing energy expenditure [19,20,21]. Overall adherence to the provided diets was reported to be good, as estimated by food consumption records, questionnaires or monitoring the return of uneaten food. 

The criteria for a low GI/GL and a high GI/GL diet differed considerably among studies; hence, no inference can be made on the effect of changing a habitual diet to a low GI/GL diet. For the low GI/GL diets the GI values ranged from 33 to 50 and the GL values from 84 to 178, whereas for the high GI/GL diets the GI values ranged from 63 to 80 and the GL values ranged from 152 to 272. The fiber content of the diets was similar in four studies [12,19,20,21] and in one study the fiber content of the low GI/GL diet was higher, primarily in insoluble fiber [14]. Consistent with the inclusion criteria the diets within each study had comparable macronutrient profiles. In the studies providing additional nutrient content information, there were no significant differences in the dietary fat type [13,14]. In one study, the cholesterol content was significantly higher in the high GI than the low GI diet [13].

An unexpected increase of fasting glucose under conditions of stable weight was observed in the low GI/GL phase compared to the high GI/GL phase, a result that seemed to be driven by the insulin resistance [13] or the high body fat content of the participants [15], respectively. In both cases, the fiber content of the low GI/GL diet was at least double that of the high GI/GL diet.

In contrast, for the parallel trials that had a weight loss component as part of the protocol and were restricted to obese individuals, the GI value of calorically restricted diets [18] or isocaloric diets accompanied with an exercise regime [19,20,21] had no effect on fasting plasma glucose concentrations. The fiber content was similar between the low and high GI diets in the latter, but not the former trial. 

Fasting insulin concentrations or %HbA1c were not significantly affected by the GI value of the diet regardless of study design or fiber content. Data for glucose or insulin concentrations measured during OGTT were not consistent across the three studies, despite similar study designs and study population characteristics [19,20,21]. Glucose and insulin AUC were reported to significantly decrease in the low GI compared to the high GI group in one study [19], but were unaffected by dietary GI in the other study [20]. The reason for the difference between the two studies is not apparent. Additionally, glucose and insulin concentrations at two hours were also not affected by the type of diet [21].

While dietary GI or GL values did not impact fasting incretin concentrations (GLP-1 and GIP), a low GL meal [16] or glucose challenge [19] suppressed the post-prandial GIP secretion after a low GI/GL diet, in agreement with lower post-prandial glucose and insulin response under the same conditions. Of note, the fiber content of the background diets was comparable between the low and high GI diet only in the latter study.

For the cross-over studies total, LDL and HDL cholesterol concentrations were affected by diet in the opposite direction; all three variables were higher after the low GI/GL diet in one trial [12], but lower after the low GI diet and not influenced by the insulin sensitivity or resistance status of participants in the other trial [14]. The authors of the first trial concluded that the improvement in total and LDL cholesterol concentrations after the high GI/GL diet may be explained by slight differences in the fatty acid profile of the diets, lower saturated and slightly higher polyunsaturated fatty acids compared to the low GI/GL diet [12]. Of note, the low GI diet of the second trial was a legume-enriched diet that provided significantly more fiber and less cholesterol than the high GI diet [14]. The discrepancy in lipid profile between the two trials may be further attributed to the wide range of GI and GL values between the respective diets, and differences in characteristics including the age and body weight. In contrast, the GI values of the diet did not appear to have a significant effect on plasma lipid concentrations in the parallel studies [18,19,21]. 

Inconsistent but significant effects were identified among studies on the basis of GI value for a number of additional CVD risk markers. However, the paucity of data for each marker precludes drawing conclusions that can be generalized. In addition to dietary fiber, as previously noted, variability in the absolute difference in dietary GI values among studies, percent of energy represented by dietary macronutrients and other factors may have contributed to the differences observed.

The inconsistency of the findings related to dietary GI/GL is also reflected by two recent meta-analyses of prospective studies identified on the relationship between GI/GL and CVD outcomes. In the first assessment, high dietary GI/GL was associated with increased CHD risk in women, but not in men, and to a greater extent in obese and overweight individuals [7]. A linear dose-response relationship was identified between GL and CHD risk in the second meta-analysis, but only a slight association of GI with CHD risk and none with stroke [22]. Of note, publication bias is becoming an increasing concern with epidemiological studies in the field of nutrition; hence great caution is an imperative when interpreting such results [23].

## 5. Conclusions

The number of available randomized controlled-feeding trials comparing low and high GI/GL diets for a minimum of four weeks that included measures of glucose homeostasis, blood lipids or inflammation is very limited. Collectively, the results of these studies are inconsistent; suggesting their use in formulating dietary recommendations is premature. This conclusion, for the most part, is consistent with prior reviews on the topic.

## Supplementary Files

Supplementary File 1 Supplementary Information (PDF, 112 KB)

## References

[1] Crapo P.A., Reaven G., Olefsky J. (1976). Plasma glucose and insulin responses to orally administered simple and complex carbohydrates. Diabetes.

[2] Crapo P.A., Reaven G., Olefsky J. (1977). Postprandial plasma-glucose and -insulin responses to different complex carbohydrates. Diabetes.

[3] Jenkins D.J., Wolever T.M., Taylor R.H., Barker H., Fielden H., Baldwin J.M., Bowling A.C., Newman H.C., Jenkins A.L., Goff D.V. (1981). Glycemic index of foods: A physiological basis for carbohydrate exchange. Am. J. Clin. Nutr..

[4] Salmeron J., Ascherio A., Rimm E.B., Colditz G.A., Spiegelman D., Jenkins D.J., Stampfer M.J., Wing A.L., Willett W.C. (1997). Dietary fiber, glycemic load, and risk of NIDDM in men. Diabetes Care.

[5] Pi-Sunyer X. (2005). Do glycemic index, glycemic load, and fiber play a role in insulin sensitivity, disposition index, and type 2 diabetes?. Diabetes Care.

[6] Franz M.J. (2008). Is there a role for the glycemic index in coronary heart disease prevention or treatment?. Curr. Atheroscler. Rep..

[7] Dong J.Y., Zhang Y.H., Wang P., Qin L.Q. (2012). Meta-analysis of dietary glycemic load and glycemic index in relation to risk of coronary heart disease. Am. J. Cardiol..

[8] Brand-Miller J., Buyken A.E. (2012). The glycemic index issue. Curr. Opin. Lipidol..

[9] Opperman A.M., Venter C.S., Oosthuizen W., Thompson R.L., Vorster H.H. (2004). Meta-analysis of the health effects of using the glycaemic index in meal-planning. Br. J. Nutr..

[10] Kelly S., Frost G., Whittaker V., Summerbell C. (2004). Low glycaemic index diets for coronary heart disease. Cochrane Database of Syst. Rev..

[11] Goff L.M., Cowland D.E., Hooper L., Frost G.S. (2013). Low glycaemic index diets and blood lipids: A systematic review and meta-analysis of randomised controlled tria. Nutr. Metab. Cardiovasc. Dis..

[12] Shikany J.M., Phadke R.P., Redden D.T., Gower B.A. (2009). Effects of low- and high-glycemic index/glycemic load diets on coronary heart disease risk factors in overweight/obese men. Metabolism.

[13] Hartman T.J., Albert P.S., Zhang Z., Bagshaw D.D., Kris-Etherton P.M., Ulbrecht J., Miller C.K., Bobe G., Colburn N.H., Lanza E. (2010). Consumption of a legume-enriched, low-glycemic index diet is associated with biomarkers of insulin resistance and inflammation among men at risk for colorectal cancer. J. Nutr..

[14] Zhang Z., Lanza E., Kris-Etherton P.M., Colburn N.H., Bagshaw D., Rovine M.J., Ulbrecht J.S., Bobe G., Chapkin R.S., Hartman T.J. (2010). A high legume low glycemic index diet improves serum lipid profiles in men. Lipids.

[15] Runchey S.S., Pollak M.N., Valsta L.M., Coronado G.D., Schwarz Y., Breymeyer K.L., Wang C., Wang C.Y., Lampe J.W., Neuhouser M.L. (2012). Glycemic load effect on fasting and post-prandial serum glucose, insulin, IGF-1 and IGFBP-3 in a randomized, controlled feeding study. Eur. J. Clin. Nutr..

[16] Runchey S.S., Valsta L.M., Schwarz Y., Wang C., Song X., Lampe J.W., Neuhouser M.L. (2013). Effect of low- and high-glycemic load on circulating incretins in a randomized clinical trial. Metabolism.

[17] Neuhouser M.L., Schwarz Y., Wang C., Breymeyer K., Coronado G., Wang C.Y., Noar K., Song X., Lampe J.W. (2012). A low-glycemic load diet reduces serum C-reactive protein and modestly increases adiponectin in overweight and obese adults. J. Nutr..

[18] Raatz S.K., Torkelson C.J., Redmon J.B., Reck K.P., Kwong C.A., Swanson J.E., Liu C., Thomas W., Bantle J.P. (2005). Reduced glycemic index and glycemic load diets do not increase the effects of energy restriction on weight loss and insulin sensitivity in obese men and women. J. Nutr..

[19] Solomon T.P., Haus J.M., Kelly K.R., Cook M.D., Filion J., Rocco M., Kashyap S.R., Watanabe R.M., Barkoukis H., Kirwan J.P. (2010). A low-glycemic index diet combined with exercise reduces insulin resistance, postprandial hyperinsulinemia, and glucose-dependent insulinotropic polypeptide responses in obese, prediabetic humans. Am. J. Clin. Nutr..

[20] Kelly K.R., Haus J.M., Solomon T.P., Patrick-Melin A.J., Cook M., Rocco M., Barkoukis H., Kirwan J.P. (2011). A low-glycemic index diet and exercise intervention reduces TNF(alpha) in isolated mononuclear cells of older, obese adults. J. Nutr..

[21] Malin S.K., Niemi N., Solomon T.P., Haus J.M., Kelly K.R., Filion J., Rocco M., Kashyap S.R., Barkoukis H., Kirwan J.P. (2012). Exercise training with weight loss and either a high- or low-glycemic index diet reduces metabolic syndrome severity in older adults. Ann. Nutr. Metab..

[22] Fan J., Song Y., Wang Y., Hui R., Zhang W. (2012). Dietary glycemic index, glycemic load, and risk of coronary heart disease, stroke, and stroke mortality: A systematic review with meta-analysis. PLoS One.

[23] Bohan Brown M.M., Brown A.W., Allison D.B. (2013). Nutritional epidemiology in practice: Learning from data or promulgating beliefs?. Am. J. Clin. Nutr..

