# Supplementary material for: Effect of Diets Differing in Glycemic Index and Glycemic Load on Cardiovascular Risk Factors: Review of Randomized Controlled-Feeding Trials
Source: Nutrients. 2013 Mar 28;5(4):1071–80. doi: 10.3390/nu5041071 (PMC3705335; doi:10.3390/nu5041071)
Supplement: Supplementary File 1 — Supplementary Information (PDF, 112 KB) [file nutrients-05-01071-s001.pdf]

# Supplementary Information

**Table S1.** Effect of low *versus* high GI/GL diets on additional markers of glucose homeostasis.

| Trial | Reference | Outcome Variables | Effect | *p*-Value |
|---|---|---|---|---|
| **1** | Shikany 2009 [12] | Insulin sensitivity index (Si) | ↔ | 0.55 |
| | | Glucose effectiveness (Sg) | ↔ | 0.36 |
| | | Acute insulin response to glucose (AIRg) | ↔ | 0.19 |
| | | Intravenous glucose tolerance (Kg) | ↔ | 0.99 |
| **2** | Hartman 2010 [13] | *C*-peptide (*n* = 64) | ↔ | 0.33 |
| | | *C*-peptide (*n* = 36 IS) | ↓ | 0.05 |
| | | *C*-peptide (*n* = 28 IR) | ↔ | 0.41 |
| **3** | Runchey 2012 [15] | Insulin-like growth factor-1 (IGF-1), (*n* = 80) | ↓ | 0.04 |
| | | IGF-1 (*n* = 29 LBF) | ↔ | 0.33 |
| | | IGF-1 (*n* = 53 HBF) | ↔ | 0.12 |
| | | IGF-binding protein-3 (IGFBP-3), (*n* = 80) | ↔ | 0.75 |
| | | IGFBP-3 (*n* = 29 LBF) | ↔ | 0.29 |
| | | IGFBP-3 (*n* = 53 HBF) | ↔ | 0.21 |
| **3** | Runchey 2012 [16] | Glucagon-like peptide (GLP-1), (*n* = 16) | ↔ | 0.50 |
| | | GLP-1 (*n* = 6 LBF) | ↔ | 0.87 |
| | | GLP-1 (*n* = 10 HBF) | ↔ | 0.25 |
| | | Glucose-dependent insulinotropic polypeptide (GIP), (*n* = 16) | ↔ | 0.22 |
| | | GIP (*n* = 6 LBF) | ↔ | 0.66 |
| | | GIP (*n* = 10 HBF) | ↔ | 0.28 |
| **5** | Solomon 2010 [19] | Fasting plasma glucose appearance rate (Basal Ra) | ↔ | 0.51 |
| | | Insulin-stimulated rate of plasma glucose disappearance (Insulin Rd) | ↔ | 0.31 |
| | | Fasting *C*-peptide | ↔ | 0.09 |
| | | Insulin resistance (IR) | ↔ | >0.05 |
| | | Fasting insulin secretion rate (Basal ISR) | ↓ | 0.02 |
| | | Oral glucose tolerance test $ISR_{AUC}$ (OGTT-$ISR_{AUC}$) | ↓ | 0.02 |
| | | OGTT-*C*-peptide | ↓ | <0.05 |
| | | OGTT-GIP | ↓ | <0.05 |
| **5** | Malin 2011 [21] | 2-h Glucose | ↔ | ≤0.39 |
| | | 2-h Insulin | ↔ | ≤0.21 |
| | | Non-esterified fatty acids (NEFA) | ↔ | ≤0.22 |
| | | % Carbohydrate utilization | ↔ | ≤0.10 |
| | | % Lipid utilization | ↔ | ≤0.10 |
| | | Clamp-glucose disposal rate divided by insulin (c-GDRI) | ↔ | ≤0.95 |
| | | c-Insulin | ↔ | ≤0.58 |
| | | c-non-oxidative glucose disposal (c-NOGD) | ↔ | ≤0.13 |
| | | c-% Carbohydrate utilization | ↔ | ≤0.98 |
| | | c-% Lipid utilization | ↔ | ≤0.98 |
| | | c-NEFA | ↔ | ≤0.12 |
| | | c -% NEFA suppression | ↔ | ≤0.06 |

Numbers in the first column indicate unique trials; publications with the same number indicate same trial; GI, glycemic index; GL, glycemic load; ↓, significantly decreased compared to high GI/GL diet; ↔, non significant effect of low compared to high GI/GL diet; IS, insulin sensitive; IR, insulin resistant; LBF, low body fat; HBF, high body fat; AUC, area under the curve.

**Table S2.** Effect of low *versus* high GI/GL diets on additional variables.

| Trial | Reference | Outcome Variables | Effect | *p*-Value |
|---|---|---|---|---|
| **1** | Shikany 2009 [12] | Tumor necrosis factor alpha receptor II (TNF-RII) | ↔ | 0.72 |
| | | Plasminogen activator inhibitor-1 (PAI-1) | ↔ | 0.93 |
| | | Fibrinogen | ↔ | 0.93 |
| **2** | Hartman 2010 [13] | Soluble TNFα receptor I (sTNF-RI), (*n* = 64) | ↔ | 0.69 |
| | | sTNFRI (*n* = 36 IS) | ↔ | 0.82 |
| | | sTNFRI (*n* = 28 IR) | ↔ | 0.75 |
| | | soluble TNFα receptor II (sTNF-RII), (*n* = 64) | ↔ | 0.58 |
| | | sTNFRII (*n* = 36 IS) | ↔ | 0.87 |
| | | sTNFRII (*n* = 28 IR) | ↔ | 0.30 |
| **3** | Neuhouser 2012 [17] | Serum amyloid A (SAA), (*n* = 80) | ↔ | 0.46 |
| | | SAA (*n* = 29 LBF) | ↔ | 0.20 |
| | | SAA (*n* = 52 HBF) | ↔ | 0.50 |
| | | Leptin (*n* = 80) | ↔ | 0.49 |
| | | Leptin (*n* = 29 LBF) | ↔ | 0.90 |
| | | Leptin (*n* = 52 HBF) | ↔ | 0.13 |
| | | Adiponectin (*n* = 80) | ↔ | 0.30 |
| | | Adiponectin (*n* = 29 LBF) | ↔ | 0.50 |
| | | Adiponectin (*n* = 52 HBF) | ↔ | 0.06 |
| **5** | Solomon 2010 [19] | Very low density lipoprotein (VLDL) cholesterol | ↔ | 0.80 |
| **5** | Kelly 2011 [20] | Mononuclear cell (MNC) TNFa | ↓ | <0.01 |
| | | MNC interleukin-6 (IL-6) | ↔ | >0.05 |
| | | Monocyte chemoattractant protein-1 (MCP-1) | ↔ | 0.07 |

Numbers in the first column indicate unique trials; publications with the same number indicate same trial; GI, glycemic index; GL, glycemic load; ↓, significantly decreased compared to high GI/GL diet; ↔, non significant effect of low compared to high GI/GL diet.

**Table S3.** Effect of low *versus* high GI/GL diets on postprandial variables of glucose homeostasis.

| Trial | Reference | Outcome variables * | Effect | *p*-Value |
|---|---|---|---|---|
| **3** | Runchey 2012 [15] | Glucose (*n* = 20) | ↓ | <0.01 |
| | | Glucose (*n* = 8 LBF) | ↔ | 0.08 |
| | | Glucose (*n* = 12 HBF) | ↓ | <0.01 |
| | | Insulin (*n* = 20) | ↓ | <0.01 |
| | | Insulin (*n* = 8 LBF) | ↓ | <0.01 |
| | | Insulin (*n* = 12 HBF) | ↓ | <0.01 |
| | | Insulin-like growth factor-1, IGF-1 (*n* = 20) | ↔ | 0.79 |
| | | IGF-1 (*n* = 8 LBF) | ↔ | 0.94 |
| | | IGF-1 (*n* = 12 HBF) | ↔ | 0.69 |
| | | IGF-binding protein-3, IGFBP-3 (*n* = 20) | ↔ | 0.15 |
| | | IGFBP-3 (*n* = 8 LBF) | ↔ | 0.79 |
| | | IGFBP-3 (*n* = 12 HBF) | ↔ | 0.11 |
| **3** | Runchey 2012 [16] | Glucose (*n* = 16) | ↓ | <0.01 |
| | | Glucose (*n* = 6 LBF) | ↓ | <0.01 |
| | | Glucose (*n* = 10 HBF) | ↓ | <0.01 |
| | | Insulin (*n* = 16) | ↓ | <0.01 |
| | | Insulin (*n* = 6 LBF) | ↓ | <0.01 |
| | | Insulin (*n* = 10 HBF) | ↓ | <0.01 |
| | | Glucagon-like peptide 1, GLP-1 (*n* = 16) | ↑ | 0.03 |
| | | GLP-1 (*n* = 6 LBF) | ↔ | 0.27 |
| | | GLP-1 (*n* = 10 HBF) | ↑ | 0.03 |
| | | Glucose-dependent insulinotropic polypeptide, GIP (*n* = 16) | ↓ | <0.01 |
| | | GIP (*n* = 6 LBF) | ↓ | <0.01 |
| | | GIP (*n* = 10 HBF) | ↓ | 0.03 |

* All values represent incremental area under the curve. Numbers in the first column indicate unique trials; publications with the same number indicate same trial; GI, glycemic index; GL, glycemic load; ↑ or ↓, significantly increased or decreased compared to high GI/GL diet; ↔, non significant effect of low compared to high GI/GL diet; LBF, low body fat; HBF, high body fat.

© 2013 by the authors; licensee MDPI, Basel, Switzerland. This article is an open access article distributed under the terms and conditions of the Creative Commons Attribution license (http://creativecommons.org/licenses/by/3.0/).
